# Haemophagocytic lymphohistiocytosis in pregnancy and the postpartum: A case series from the national HLH network

**DOI:** 10.1177/1753495X251356108

**Published:** 2025-07-08

**Authors:** Bethan Goulden, Eleanor Singer, Benjamin Bennett, Eman Elfar, Kazi Fardeen, Ian Giles, Elizabeth Rankin, Joanna Girling, Harry Suzuki, Kate Wiles, Maria Mouyis, Rachel Tattersall, Alexis Jones, Jessica Manson

**Affiliations:** 1 Department of Ageing, Rheumatology and Regenerative Medicine, 4919 University College London, UK; 2 Department of Infectious Diseases, 473300 Queen Elizabeth University Hospital, UK; 3 Department of Rheumatology, 9762 West Middlesex University Hospital, Chelsea Westminster Hospital NHS Foundation Trust, UK; 4 Department of Rheumatology, 111990 Kings College London Hospital, UK; 5 Department of Rheumatology, 158987 Royal Free Hospital, UK; 6 Department of Rheumatology, University Hospitals Birmingham, UK; 7 Department Obstetrics and Gynaecology, 9762 West Middlesex University Hospital, Chelsea Westminster Hospital NHS Foundation Trust, UK; 8 Department of Gastroenterology, 473300 Queen Elizabeth University Hospital, UK; 9 Department of Women's Health, 9744 Barts Health NHS Trust, UK; 10 Department of Rheumatology, 575329 Bedfordshire NHS Foundation Trust, UK; 11 Department of Rheumatology, Royal Hallamshire Hospital, 7318 Sheffield Teaching Hospitals NHS Foundation Trust, UK; 12 Department of Rheumatology, 98548 University College London Hospital, UK

**Keywords:** Pregnancy, postpartum, haemophagocytic lymphohistiocytosis, macrophage activation syndrome, anakinra, SAMD9L

## Abstract

Haemophagocytic lymphohistiocytosis (HLH) is a hyperinflammatory sepsis-like syndrome that accounts for 1% of maternal deaths in the United Kingdom (UK). In 2019, a UK-wide HLH network was developed to provide specialist advice for patients with HLH. Until September 2024, eight individuals had been referred to this service with HLH onset during pregnancy or within 6 months postpartum, and this article summarises their management. Shared themes were of postpartum predominance, with onset typically within a month of delivery, preceding infection, and underlying immune dysfunction. Common therapies included corticosteroids and the interleukin-1 receptor antagonist, anakinra. Most individuals required level 3 care, three were considered for extracorporeal membrane oxygenation, and one died. HLH should be included in the differential of maternal sepsis, given all cases presented with fever and organ dysfunction, particularly if there is ongoing deterioration despite antimicrobial therapy and/or without an identified source.

## Introduction

**Figure 1.**
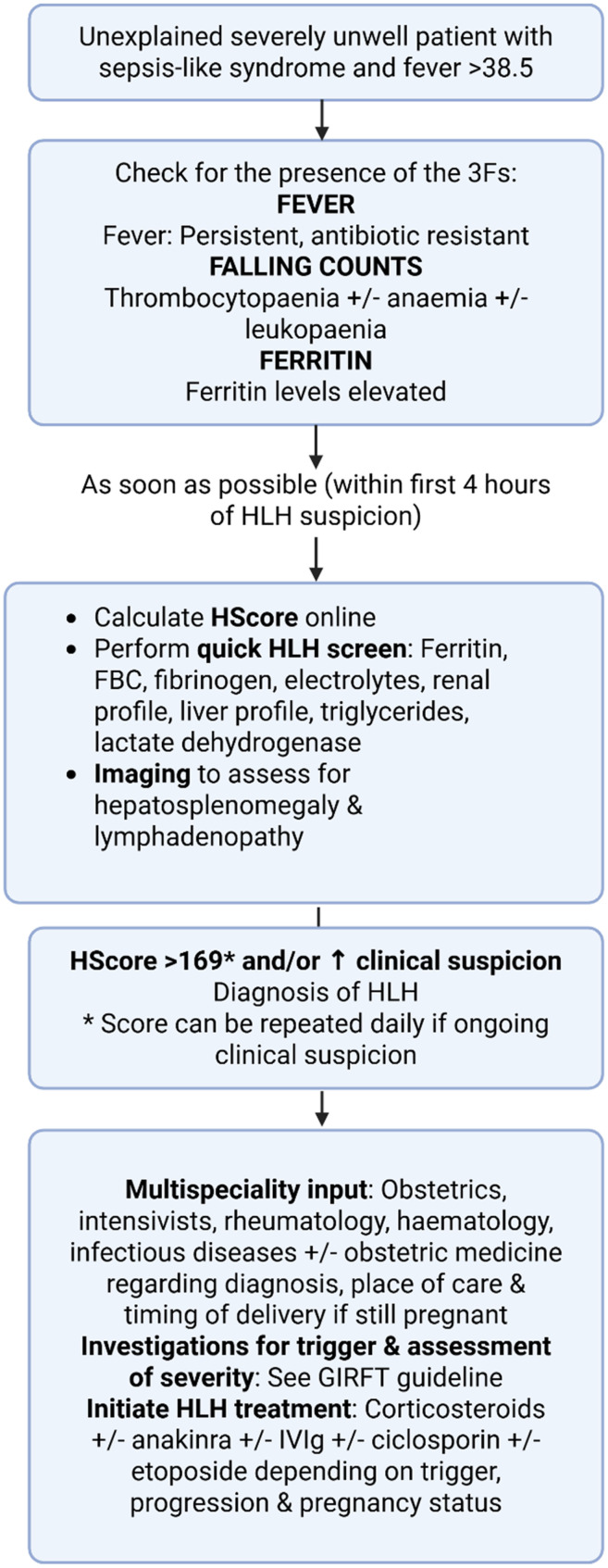
Summary of the recognition and management of HLH – adapted with permission from the Getting It Right First Time (GIRFT) HLH guideline.^3^ FBC = full blood count, GIRFT = getting it right first time, HLH = haemophagocytic lymphohistiocytosis, IVIg = intravenous immunoglobulin.

Haemophagocytic lymphohistiocytosis (HLH) is a hyperinflammatory sepsis-like syndrome which typically presents with fever, cytopaenia and progressive multi-organ dysfunction.^1^ Individuals with HLH may have accompanying rash, lymphadenopathy, hepatosplenomegaly, and/or neurological dysfunction.^1^ Hyperferritinaemia is a core feature of the disease, but other abnormalities include cytopaenias (particularly thrombocytopaenia), elevated liver transaminases, hypertriglycerideaemia, and hypofibrinogenaemia.^1^ Consensus guidelines from the hyperinflammation and HLH across speciality collaboration (HiHASC), recommend the use of the 3 Fs for screening for HLH in the unwell patient – Fever, Falling cell counts, and raised Ferritin.^2^ If present, further investigations should be undertaken to confirm or exclude HLH, including measurement of the HScore and evaluation for HLH triggers.^2–5^ See Figure 1 for a summary of the recognition and management of HLH.

HLH in adults is often termed *secondary HLH* as it develops secondary to autoimmune or autoinflammatory disease (e.g. Still's disease, systemic lupus erythematosus/SLE, inflammatory bowel disease), malignancy (particularly lymphoma), and/or infection (e.g. Epstein Barr virus/EBV, cytomegalovirus/CMV, herpes simplex virus/HSV).^1,2^ Primary HLH, due to underlying genetic defects, is most commonly a paediatric onset disease; however, there is increasing recognition that genetic abnormalities may also be causative or contributory to a significant number of patients with adult-onset HLH.^6^

The United Kingdom (UK) confidential enquiry into maternal deaths (MBRRACE-UK) reported eight maternal deaths secondary to HLH between 2013 and 2021 – two in individuals from white British/Scottish/Irish ethnicities and the remaining six from global majority ethnicities.^7–9^ This finding equates to HLH being the cause of around 1% of UK maternal deaths. Given the under recognition of HLH and its similarity to sepsis, we hypothesise that the true prevalence is likely to be higher.

## Case series

The UK national HLH network was established in 2019, and between January 2022 and September 2024, eight patients were referred with HLH onset during pregnancy or within 6 months postpartum. Individual patient consent was obtained for inclusion in the case series. Details of the index pregnancies associated with HLH are summarised in Table 1.

**Table 1.** Patient summaries.

Patient	Pregnancy outcome	Mode of delivery	Infant outcome	Obstetric complications	Timing of HLH symptom onset	HLH triggers	Abnormal serology* /cellular assays	Treatment	Complications
1	Miscarriage <12w	n/a	n/a	n/a	98d PP (but 6d following ERPC)	New Δ AOSD, retained products of conception	Nil	Steroids Anakinra	ICU, AKI
2	Livebirth at 38w3d	C-section	28^th^ centile	Induced (slowing of fetal growth)	2^nd^ trimester	New Δ SLE, preceding covid-19	Anti-Ro, anti-La, RF	Steroids HCQ	Nil
3	Livebirth at 38w3d	C-section	8^th^ centile	Induced (maternal hypertension)	19d PP	C-section wound cellulitis	Anti-Ro	Steroids Anakinra	ICU, referred for ECMO, AKI, MI post-recovery
4	Livebirth at 39w2d	C-section	12^th^ centile	Induced	49d PP	Colitis & intra-abdominal collection Pseudomonas bacteraemia	Anormal granule release assay, repeat normal ANA positive but no ENA measured	Steroids Anakinra	ICU, AKI
5	Livebirth at 39w0d	C-section	51^st^ centile	Nil	12d PP	E.coli UTI	Anti-Ro, anti-La, RF	Steroids Anakinra IVIg Rituximab Etoposide	ICU, referred for ECMO, AKI, LVEF nadir 35%, died 8 months PP
6	Livebirth at 39w1d	C-section	43^rd^ centile	Nil	7d PP	Known SS, possible intra-abdominal infection	Anti-Ro, anti-La, RF	Steroids Anakinra	ICU, cardiac arrest, LVEF nadir 15% (now normalised), ECMO
7	Livebirth at 25w0d	C-section	3^rd^ centile, trisomy 21	Pre-eclampsia	3d PP	Known SLE & immune TTP; new Δ obstetric APS	Anti-RNP, anti-Sm, triple positive aPLs, low perforin, SAMD9L mutation	Steroids Anakinra IVIg Rituximab	AVN of the hip post-recovery
8	Livebirth at 31w3d	C-section	1^st^ centile	Pre-eclampsia	16d PP	E.coli UTI, c-section wound cellulitis, new Δ obstetric APS	Triple positive aPLs	Steroids Anakinra	ICU

AKI = acute kidney injury, AOSD = adult-onset still's disease, ANA = anti-nuclear antibody, aPLs = anti-phospholipid antibodies (anti-cardiolipin, anti-β2 glycoprotein I, lupus anticoagulant), APS = anti-phospholipid antibody syndrome, AVN = avascular necrosis, C-section = caesarean section, d = days, ECMO = extracorporeal membrane oxygenation, ENA = extractable nuclear antigens, ERPC = evacuation of retained products of conception, HCQ = hydroxychloroquine, ICU = Intensive Care Unit admission, LVEF = left ventricular ejection fraction, MI = myocardial infarction, PP = postpartum, RF = rheumatoid factor, SLE = systemic lupus erythematosus, SS = Sjogren's syndrome, TTP = thrombotic thrombocytopaenic purpura, UTI = urinary tract infection, w = weeks, Δ = diagnosis, * = samples taken prior to any IVIg administration; centile calculated using Fetal Medicine Foundation calculator.^10^

Median age was 30.5 years (range 23–41 years old). Ethnicities were Asian (*n* = 4), White (*n* = 2) and Black (*n* = 2). HLH was associated with 1^st^ (*n* = 3), 2^nd^ (*n* = 2) and 3^rd^ (*n* = 3) pregnancies. None were smokers. Median BMI was 28 kg/m^2^ (range 21–40 kg/m^2^). One patient had consanguineous parents. Among the five with a prior pregnancy, all had experienced previous obstetric complications, including miscarriage (*n* = 1), stillbirth associated with congenital anomalies (*n* = 1), small for gestational age (*n* = 2), and preterm birth (*n* = 1).

Two had pre-existing autoimmune rheumatic diseases – one with juvenile SLE and immune thrombotic thrombocytopaenic purpura (TTP), another with Sjogren's syndrome. Other significant co-morbidities included hypothyroidism (patient 1), keratitis and severe asthma (patient 2), undifferentiated colitis (patient 4), Chiari malformation with ventriculoperitoneal shunt (patient 5), and hypertension (patient 3). Two had a history of sepsis; one of which was postpartum in a prior pregnancy (patient 3) and one non-pregnancy related episode, which required level 3 care (patient 5).

Presentation was with fever (100%), rash (50%), diarrhoea or abdominal pain (50%), and arthralgia (25%). Median nadir platelet count was 30 × 10^9^/L (range 7 × 10^9^/L to 87 × 10^9^/L), median peak ferritin 9590.5 µg/L (range 5964–71,748 µg/L), median peak alanine transaminase 222 IU/L (range 57–454 IU/L), and median peak triglycerides 7.3 mmol/L (range 3.76–15 mmol/L). Of five people who underwent deep skin biopsies, none showed features of intravascular lymphoma. Bone marrow biopsy was performed in seven, with haemophagocytosis confirmed in five. At presentation, five had a documented HScore which ranged from 187 (70–80% probability of HLH) to 243 (>99% probability of HLH).

Patient 2 developed HLH during the 2^nd^ trimester of pregnancy, but all others became unwell postpartum – one following a miscarriage with delayed evacuation of retained products of conception, and a further six following birth by Caesarean section. Of the seven developing HLH postpartum, median time to HLH presentation from delivery was 16 days (range 3–49 days). Both preterm deliveries occurred in the context of persistent triple positive anti-phospholipid antibodies and severe early onset pre-eclampsia (<34 weeks), both therefore meeting criteria for the obstetric anti-phospholipid antibody syndrome (APS).^11^ The presence of these antibodies were known prior to delivery in patient 7. Apart from placental infarcts, neither had evidence of macro- or microthrombosis indicative of the alternative diagnosis, catastrophic APS. In addition to the two patients with existing diagnoses of autoimmune rheumatic diseases, one received a new diagnosis of Still's disease, and one received a new diagnosis of SLE. Two individuals had no clinical evidence of an autoimmune rheumatic disease but had positive serology (e.g. anti-Ro, anti-La, rheumatoid factor), and two had evidence on cellular assays of abnormalities which prompted consideration of primary HLH. The abnormal granule release assay in one individual normalised on repeat. The individual with low perforin had a negative HLH genetic panel but was ultimately diagnosed with a truncating mutation of SAMD9L following investigations for prolonged cytopaenias, suggestive of a possible underlying SAMD9L-associated autoinflammatory disease.^12^

Confirmed or suspected preceding infection was identified in seven out of eight, including Caesarean section wound cellulitis, urinary tract infection, infection associated with retained products of conception, and COVID-19. No episodes of HLH were triggered by malignancy, and following extensive screening for a broad range of pathogens in all individuals, no cases of tuberculosis, human immunodeficiency virus, CMV, EBV or HSV were identified.

All received steroids, seven of eight received anakinra, two were treated with intravenous immunoglobulin (IVIg) and rituximab, and one received etoposide. Alongside immunosuppression, all received antibiotics, 7/8 prophylactic antiviral therapy with acyclovir, and 6/8 prophylactic antifungal therapy.

Six required level 3 care, three were referred for extracorporeal membrane oxygenation, one survived a cardiac arrest, and one mother died 8 months postpartum, presumed secondary to infective complications. In the months after HLH, one individual had a myocardial infarction, and another developed hip pain and was ultimately diagnosed with bilateral avascular necrosis of the hips.

One post-HLH pregnancy has occurred among these individuals. The mother continued anakinra throughout and delivered her infant, at term, without recurrence of HLH.

## Conclusions

Common themes in this series of individuals with pregnancy-associated HLH were: postpartum predominance, preceding infection, evidence of underlying autoimmunity and immune dysfunction. All individuals with prior pregnancy had had a previous adverse pregnancy outcome, but one post-HLH pregnancy treated with anakinra has been uncomplicated and without recurrent HLH. All presented with fever and were initially presumed to have bacterial sepsis. Most required level 3 care and were managed with anakinra and corticosteroids.

A single-centre experience of pregnancy-associated HLH from China, and a UK-based series have been previously reported.^13,14^ The Chinese series included 13 individuals, whilst the British one included details of 5 maternal deaths previously reported via MBRRACE-UK, alongside details of two survivors. Only the UK series included postpartum HLH, incorporating one individual with HLH 11 months following delivery. Underlying drivers reported in these series included infection (EBV, CMV, parvovirus B19), rheumatic disease (SLE, Still's disease, inflammatory arthritis), and sickle cell crisis; in seven of 20 cases, the cause was unknown. The predilection for the postpartum in our series is therefore novel but may reflect the lack of inclusion of this timepoint in previous work, and a failure to consider recent pregnancy as relevant to a later presentation of HLH outside of obstetric services.

In all populations, HLH recognition, diagnosis and treatment are complex and require multi-speciality input.^2,3,15^ Pregnancy and the immediate postpartum period further complicate this process. Physiological changes in cell counts can make recognition of HLH-driven cytopaenias problematic – platelet counts and haemoglobin fall in pregnancy secondary to haemodilution, whilst neutrophil counts increase.^16^ Triglyceride concentrations can triple, and fibrinogen concentrations increase.^16^ The HScore is not validated in pregnancy or the postpartum period and must be interpreted in the context of gestational physiology. Furthermore, the immune adaptations to pregnancy predispose to severe infections, such as HSV, which phenotypically overlaps with HLH (e.g. fever, transaminitis) and has the potential to evolve into HLH in some individuals.^17^

Corticosteroids and anakinra were the most commonly used immunomodulatory agents in this series. Corticosteroids are a first-line therapy in HLH due to their widespread availability, onset of action and cost.^18^ Non-fluorinated steroids (e.g. methylprednisolone, prednisolone) are metabolised by placental 11β-hydroxysteroid dehydrogenase, resulting in minimal transplacental passage.^19^ Other agents utilised in HLH which are compatible with pregnancy include IVIg and ciclosporin.^19^

The biologic medication, anakinra, has developed widespread acceptance in HLH management in recent years.^18^ Given that HLH often presents with a sepsis-like syndrome and that HLH may itself be driven by infection, anakinra benefits from having previously been trialled in sepsis.^20^ In a randomised controlled trial, anakinra use did not increase the incidence of adverse reactions, microbial superinfections, or mortality, and a survival benefit was seen among participants with HLH-like features.^20,21^ Anakinra is a recombinant protein analogue of the naturally occurring protein, interleukin-1 receptor antagonist. Given its large molecular weight and the lack of an Fc segment – minimal transplacental transfer of anakinra is expected, and preclinical data in rats and rabbits, at doses up to 100 times the human dose, are reassuring – with no adverse effects seen on fertility, pregnancy or teratogenicity.^22^ 48 human pregnancy exposures, summarised in the latest British Society for Rheumatology (BSR) guideline on prescribing in pregnancy and lactation, do however highlight two cases of renal anomalies amongst in-utero exposed infants – one to a mother with Still's disease, one in a twin pregnancy to a mother with an hereditary autoinflammatory syndrome.^23,24^ Two further cases of oligohydramnios (which may be associated with fetal renal dysfunction) were also observed.^25^ No causal link between anakinra and these anomalies have been made, and, given the life-threatening consequences of HLH to mother and fetus, anakinra should not be withheld in the setting of HLH. Therefore, the BSR guideline states that anakinra ‘may be considered to manage severe maternal disease in pregnancy if no other pregnancy-compatible drugs are suitable’.^19^ With regard to lactation-compatibility, guidelines support the use of corticosteroids, IVIg, ciclosporin and anakinra, in breastfeeding.^19,26^

Finally, it is well established that the postpartum period predisposes to flare in inflammatory conditions such as SLE and rheumatoid arthritis^27,28^ – we hypothesise that the immunological transition from pregnancy to postpartum may also predispose to hyperinflammation. An alternative, or perhaps complementary, hypothesis is that postpartum HLH reflects a form of fetal graft versus host disease (GVHD), as proposed in a case report published in the New England Journal of Medicine (NEJM) in 2023.^29^ In the reported case, fetal GVHD was diagnosed due to the clinical similarities with GVHD (fever, rash and diarrhoea), and the identification of fetal cells in bone marrow and skin.^29^ Whilst we did not have access to testing that would allow fetal cell identification, these clinical features (i.e. fever, rash, diarrhoea) were also common in our series. However, bidirectional trafficking of fetal and maternal cells across the placenta occurs throughout all pregnancies, particularly around the time of delivery.^30^ Fetal microchimeric cells are found in peripheral blood, healthy and injured maternal tissues, both immediately and many years after pregnancy.^30^ The patient described in the NEJM would have met criteria for HLH, was hyperferritinaemic, had haemophagocytosis on her marrow, and recovered following treatment with corticosteroids and IVIg.^29^ Historically, HLH induced by rheumatic disease was termed macrophage activation syndrome, but modern guidelines now advocate that HLH is called HLH, regardless of trigger, to facilitate collaborative learning across the multiple disciplines involved in HLH care clinically and academically.^2^ We argue that the field of HLH has benefited from this shared language. Postpartum HLH should continue to be referred to as such, rather than introducing novel terms such as fetal GVHD, with the pathogenic role of fetal microchimeric cells considered a promising avenue of future research in pregnancy-associated and postpartum HLH.

When assessing the critically unwell febrile patient, sepsis should remain the primary concern. In those who continue to deteriorate despite antimicrobial therapy or without recognised cause, the evaluation should also consider occult, resistant, and non-bacterial infections (i.e. viral, mycobacterial).^17^ Alongside this approach, clinicians must also examine for features of non-infective inflammatory conditions, including TTP and HLH, as endorsed by maternal sepsis guidelines.^17^

This case series adds to the growing body of literature exploring pregnancy-associated HLH. Prospective research and educational initiatives are now required to expand our understanding of incidence, triggers, and outcomes in the peripartum period. A 5-year, prospective, UK-wide study of HLH in pregnancy using the UK Obstetric Surveillance System platform is currently active and expected to report in 2030.^31^
